# The role of gallery forests in maintaining Phlebotominae populations: potential *Leishmania* spp. vectors in the Brazilian savanna

**DOI:** 10.1590/0074-02760170126

**Published:** 2017-10

**Authors:** Tâmara Dias Oliveira Machado, Thaís Tâmara Castro Minuzzi-Souza, Tauana de Sousa Ferreira, Luciana Pereira Freire, Renata Velôzo Timbó, Tamires Emanuele Vital, Nadjar Nitz, Mariana Neiva Silva, Alcinei de Souza Santos, Nathyla Morgana Cunha Sales, Marcos Takashi Obara, Andrey José de Andrade, Rodrigo Gurgel-Gonçalves

**Affiliations:** 1 Universidade de Brasília, Faculdade de Medicina, Área de Patologia, Laboratório de Parasitologia Médica e Biologia de Vetores, Brasília, DF, Brasil; 2 Instituto Federal de Educação, Ciência e Tecnologia do Tocantins, Coordenação de Ciências Matemáticas e Naturais, Palmas, TO, Brasil; 3 Universidade de Brasília, Faculdade de Medicina, Área de Patologia, Laboratório Interdisciplinar de Biociências, Brasília, DF, Brasil; 4 Universidade de Brasília, Ceilândia, DF, Brasil; 5 Universidade Federal do Paraná, Setor de Ciências Biológicas, Departamento de Patologia Básica, Curitiba, PR, Brasil

**Keywords:** Nyssomyia whitmani, Lutzomyia longipalpis, Leishmania amazonensis, Trypanosoma sp., Crithidia fasciculata

## Abstract

**BACKGROUND:**

Knowledge on synanthropic phlebotomines and their natural infection by *Leishmania* is necessary for the identification of potential areas for leishmaniasis occurrence.

**OBJECTIVE:**

To analyse the occurrence of Phlebotominae in gallery forests and household units (HUs) in the city of Palmas and to determine the rate of natural infection by trypanosomatids.

**METHODS:**

Gallery forests and adjacent household areas were sampled on July (dry season) and November (rainy season) in 2014. The total sampling effort was 960 HP light traps and eight Shannon traps. Trypanosomatids were detected in Phlebotominae females through the amplification of the SSU rDNA region, and the positive samples were used in ITS1-PCR. Trypanosomatid species were identified using sequencing.

**FINDINGS:**

A total of 1,527 sand flies representing 30 species were captured in which 949 (28 spp.) and 578 (22 spp.) were registered in July and November, respectively. In July, more specimens were captured in the gallery forests than in the HUs, and *Nyssomyia whitmani* was particularly frequent. In November, most of the specimens were found in the HUs, and again, *Ny. whitmani* was the predominant species. *Lutzomyia longipalpis* was commonly found in domestic areas, while *Bichromomyia flaviscutellata* was most frequent in gallery forests. Molecular analysis of 154 pools of females (752 specimens) identified *Leishmania amazonensis*, *L*. *infantum*, and *Crithidia fasciculata* in *Ny. whitmani*, as well as *L*. *amazonensis* in *Lu*. *longipalpis*, *Trypanosoma* sp. and *L*. *amazonensis* in *Pintomyia christenseni*, and *L*. *amazonensis* in both *Psathyromyia hermanlenti* and *Evandromyia walkeri*.

**MAIN CONCLUSIONS:**

These results show the importance of gallery forests in maintaining Phlebotominae populations in the dry month, as well as their frequent occurrence in household units in the rainy month. This is the first study to identify *Leishmania*, *Trypanosoma*, and *Crithidia* species in Phlebotominae collected in Palmas, Tocantins, Brazil.

The leishmaniases are diseases caused by protozoans of the genus *Leishmania* (Kinetoplastida: Trypanosomatidae) that are transmitted by several phlebotomine species (Diptera: Phlebotominae) comprising an important public health problem worldwide. In endemic areas, phlebotomine species are commonly found in both the sylvatic and domestic environments, and their natural infection rates by *Leishmania* spp. are generally low (Paiva et al. 2006, Pita-Pereira et al. 2011).

The main factors that contribute to the proximity of phlebotomine species to domestic environments are urbanisation, deforestation and host availability (Quinnell & Dye 1994, Vilela et al. 2011). These anthropogenic changes produce landscapes in which households are close to remaining forest patches where the enzootic cycles of *Leishmania* species are present. Phlebotomine populations can spread from these forests and colonise human environments. Thus, detecting natural infection in Phlebotominae in environments with different degrees of human modification is important for epidemiological studies on leishmaniasis.

Rapid local socioeconomic growth after the creation of the Brazilian state of Tocantins in 1988, associated with human migration, deforestation, and the poor quality or lack of sanitation systems, are all examples of human modifications. Recently, this region has experienced environmental changes resulting from agricultural activities and hydroelectric power plants, both of which are conducive to the spread of certain phlebotomine species to human environments and which have contributed to leishmaniasis outbreaks (Vilela et al. 2011). Approximately 70 sand fly species have been recorded in Tocantins (Andrade-Filho et al. 2001, Vilela et al. 2011, 2013, Machado et al. 2012, Godoy et al. 2017), some of which (*Nyssomyia whitmani*, *Lutzomyia longipalpis*, *Evandromyia bourrouli*, *Ny. antunesi*, *Micropygomyia villelai*, *Psychodopygus complexus*, and *Ps. ayrozai*) have been recorded more frequently in modified environments (Machado et al. 2012, Vilela et al. 2013, Godoy et al. 2017). *Ps. complexus* and *Ps. ayrozai* have been found to be infected by *Leishmania* (*Viannia*) *braziliensis* in the state (Vilela et al. 2013).

The construction of Palmas, the capital of Tocantins, turned large swaths of the Cerrado biome into fragments of forest distributed throughout the city. Certain sand fly species have been able to occupy these modified habitats, which could favor the circulation of *Leishmania* species in domestic environments. High rates of *Leishmania* infection in dogs and children in other cities in Tocantins are evidence of this process (Bigeli et al. 2012, Teles et al. 2012, Oliveira et al. 2014). Between 2011 and 2015, 1,473 new cases of human visceral leishmaniasis (VL) and 2,685 new cases of American cutaneous leishmaniasis (ACL) were confirmed in Tocantins. One hundred and eleven of the VL cases and 169 of the ACL cases were registered in Palmas (data obtained in cooperation with the local Secretariats of Health). These results reflect the importance for more active surveillance, including monitoring of occurrences and of natural phlebotomine infection in cities in this state.

Knowledge on phlebotomine species that are more likely to adapt to human environments and on the levels of natural infection by *Leishmania* is necessary for the identification potential areas for leishmaniasis occurrence. Though sand fly species have been detected in the Taquaruçu district of Palmas (Machado et al. 2012), there are no records of the phlebotomine fauna in the remaining gallery forests in the city, nor information about their natural infection by *Leishmania* species. Therefore, the present study sought to analyse the occurrence of sand fly species in gallery forests and household units (HUs) in downtown Palmas and the Taquaruçu district specifically in dry and wet months, and also to evaluate their rate of natural infection by *Leishmania* spp. and other trypanosomatids in these areas.

## MATERIALS AND METHODS

**Fig. 1:**
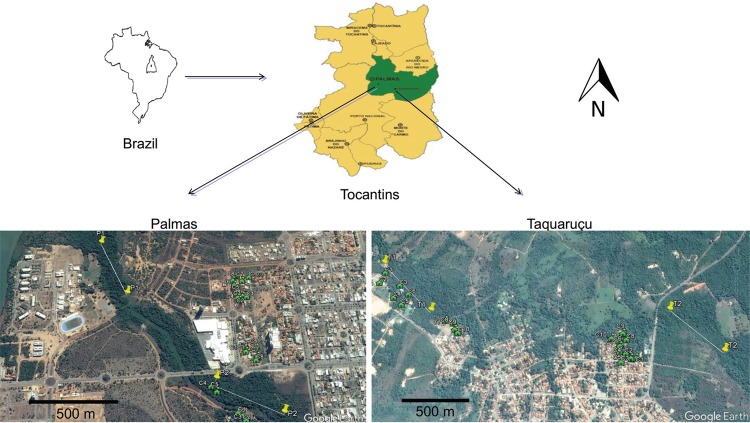
: location of study areas. The yellow pins connected by lines indicate points of reference for sampling trails in the gallery forests, and the green symbols represent the household units (HUs) sampled in downtown Palmas and the Taquaruçu district. Source: Google Earth 2016.

*Study area* - Palmas is located in the central region of the Brazilian state of Tocantins (10º09’48”S, 48º21’04”W, Fig. 1). Palmas has a humid-subhumid climate, with an average annual temperature of 28ºC and average annual rainfall of 1,600-1,700 mm. The population is estimated to be 272,726 residents, and the city covers 2,218.943 km^2^ (IBGE 2013). Vegetation types range from the savanna known as the Cerrado *sensu strictu*, grasslands, montane savanna, riparian forest, and gallery forest. The district of Taquaruçu (10º10’33’’S, 48º03’57’’W) is located in a rural area of Palmas (Fig. 1) within the Serra do Lajeado Environmental Protection Area and has an area of 639km^2^. In 2010, the population of this district was 4,739 residents (http://polis.org.br). Its climate is the same as that of Palmas, with an average annual temperature of 27ºC and average annual rainfall of 1,600 mm. Vegetation includes the Cerrado *sensu strictu*, the savanna known as the Palmeiral, and gallery forests.

*Phlebotominae collection and identification* - Sand flies were collected in downtown Palmas and in the Taquaruçu district from two gallery forests in each area and 10 HUs adjacent to each gallery forest (Fig. 1). HP light traps and Shannon traps were used in the collections. The HP traps were installed at a height of approximately 1.5 m and were exposed for 12 h starting at sunset, and the Shannon traps were left in place for four hours (18:00 - 22:00). Twenty HP traps were placed in each gallery forest in linear transects approximately 500 metres in length. Two HP traps were installed in each household: one in a peridomestic location (such as an animal housing structure), and one inside the household. At each HP trap site, three collections were performed on three consecutive sampling days. One capture with a Shannon trap was performed in each gallery forest. Sampling in these areas was repeated in two months (the dry season in June and the rainy season in November 2014). There were 15 sampling days in each month. Total sample efforts were represented by 960 HP traps (50% of which were installed in the gallery forests) and eight Shannon traps (100% of which were installed in the gallery forests).

After screening, males and females were euthanised and placed in Eppendorf tubes with 70% alcohol. The males were clarified and mounted in Canada balsam between the slide and the coverslip. The females were dissected before being mounted on the slides. The head and final portion of the abdomen (the last three segments) were separated from the body following the same clarification procedure used for the males. The structures were mounted for specific identification. The Phlebotominae were identified following Galati (2003, 2016) and genera abbreviations were used in accordance with Marcondes (2007). Some specimens were identified only at the subtribe, genus or subgenus level due to the condition of the material.

*DNA Extraction and polymerase chain reaction (PCR)* - Parts of the females’ abdomens were grouped into pools of up to 10 specimens after species identification, capture site, and date of collection. Each pool was transferred to a sterile, 1.5 mL Eppendorf tube for DNA extraction using the Illustra™ Tissue and Cells Genomic Prep Mini Spin Kit (GE Healthcare). The samples remained in proteinase K at 37ºC overnight, and the following steps were performed according to the manufacturer’s instructions. To determine the quality of the DNA extracted, the cacophony gene of the IVS6 region of Phlebotominae was detected using specific primers: Llcac5’-GTGGC-CGAACATAATGTTAG-3’ and Llcac 5’-CCACGAACAAGT-TCAACATC-3’ (Lins et al. 2002). The amplification reaction was performed in a final volume of 25 μL containing 1X reaction buffer (20 mM Tris-HCl, pH 8.4, 50 mM KCl; Invitrogen), 1.0 mM MgCl_2,_ 0.2 μM dNTPs solution (GE Healthcare), 0.5 μM of each primer, 1.5 U of Invitrogen Platinum Taq DNA polymerase (Life Technologies, Brazil), and 3μL of DNA (5 ng/μL). The reactions were performed in the MyCycler™ thermal cycler system (Bio-Rad) with the following program: 5 min at 95ºC, followed by 35 cycles at 95ºC for 30 s, 57ºC for 30 s, 72ºC for 30 s, and a final extension for 10 min at 72ºC. The positive control (Phlebotominae DNA) and the blank sample (no DNA) were included. The amplified products were separated by electrophoresis in 1.3% agarose gel stained with ethidium bromide at 0.5 μg/mL and in TAE buffer (90 mM Tris-acetate at pH 8.0; 25 mM EDTA) and visualised using an Alpha Imager R Mini System (Alpha Innotech).

To detect Phlebotominae infection by trypanosomatids, PCRs were performed using the S4 (5’-GAT CCA GCT GCA GGT TCA CC-3’) and S12 (5’-GGT TGA TTC CGT CAA CGG AC-3’) primers to amplify the SSU rDNA region (Uliana et al. 1994). The PCR was prepared in a final volume of 25 µL containing 1X reaction buffer (20 mM Tris-HCl, pH 8.4, 50 mM KCl; Invitrogen), 1.5 mM MgCl_2_, 0.2 μM dNTPs solution (GE Healthcare), 0.75 μM of each primer, 1.5 U of Invitrogen Platinum Taq DNA polymerase (Life Technologies, Brazil), and 3 μL of DNA (5 ng/μL) from the samples. The reactions were realised in the thermocycler BIO-RAD™ MyCycler and the follow conditions were used: 94ºC for 3min, followed by 35 cycles at 94ºC for 1 min, 58ºC for 1 min, 72ºC for 1 min, and a final extension in 72ºC for 7 min. The amplified products were separated by electrophoresis as described above.

After the dilution of the product of the first SSU-PCR (amplifying a 520-bp fragment), nested PCR was performed using the specific primers S17 (5´-CCA AGC TGC CCA GTA GAAT-3´) and S18 (5´-TCG GGC GGA TAA AAC CC-3´) (Savani et al. 2009), under the same conditions as those used in the first reaction. The product of the first PCR was diluted in water in the proportion 1:10 and 2 μL were used in the nested PCR. This PCR was prepared in a final volume of 25 μL containing of 1X reaction buffer (20 mM Tris-HCL pH 8.4, 50 mM KCl, Invitrogen), 0.2 μM dNTPs solution (GE Healthcare), 1.5 mM MgCl_2_, 0.75 μM of each primer, and 1.5 U Invitrogen Platinum Taq DNA polymerase (Life Technologies, Brazil). PCR products were separated by 1.3% agarose gel electrophoresis as described above. The positive controls used in these reactions were DNA samples from the parasites extracted from laboratory cultures: *L. braziliensis* (Lb), *L. infantum* (Li) or synonym *L. chagasi* (Lc), and *Trypanosoma cruzi* (Tc). The negative controls were DNA from uninfected mice.

The samples that showed positive amplicons in the nested SSU-PCR were tested in PCR using the ITS region of the ribosomal RNA gene (ITS1), specific for *Leishmania* spp., producing a fragment that ranged from 302 to 338-bp in length. The primers used were LITS1 (5’ CTG GAT CAT TTT CCG ATG 3’) and L5.8S (5’ TGA TAC CAC TTA TCG CAC TT 3’) (El Tai et al. 2000). The amplification reaction was performed in a final volume of 25 μL containing 1X reaction buffer (20 mM Tris-HCL, pH 8.4, 50 mM KCl, Invitrogen), 1.5 mM MgCl_2_, 0.2 μM dNTPs solution (GE Healthcare), 0.25 μM of each primer, 1.5 U of Invitrogen Platinum Taq DNA polymerase (Life Technologies, Brazil), and 5 μL of DNA (20 ng/μL). The reactions were performed in the MyCycler™ thermal cycler system (Bio-Rad) with the following program: 95ºC for 5 min, 35 cycles at 95ºC for 30 s, 58ºC for 30 s, 72ºC for 30 s, and a final extension for 5 min at 72ºC. The positive and negative controls were the same used in the SSU-PCR, with the exception of *T. cruzi.* The product of this PCR was reamplified with the same primers, and 2 µL of the product from the first reaction was used as template. The same conditions were kept for the reamplification. The amplified products were separated by electrophoresis as described above.

*Sequencing* - The fragments amplified in all PCRs were purified using an Illustra™ GFX PCR DNA and Gel Band Purification Kit (GE Healthcare, New York, USA) and sequenced to identify the *Leishmania* species and other trypanosomatids. The sequencing was performed at the Human Genome and Stem-Cell Research Center of the University of São Paulo (USP). The sequences were edited in the Geneious software (Biomatters, New Zealand) and were then compared to the sequences from *Leishmania* species and other trypanosomatids deposited in GenBank using the basic local alignment search tool (BLAST) algorithm from the National Center for Biotechnology Information (NCBI). The parasites were identified considering 99%-100% of identity with the sequences deposited in GenBank.

*Data analysis* - Fisher’s exact tests were performed using the GraphPad software to determine the differences in Phlebotomine species frequency between the habitats (gallery forests versus HUs), months (the dry month in July versus the rainy month in November), and locations (Palmas versus Taquaruçu). Differences in the proportions of phlebotomine pools positive for *Leishmania* between habitats and locations were also determined using Fisher’s exact test. The differences were considered statistically significant when p < 0.05.

## RESULTS

A total of 1,527 Phlebotominae specimens (738 from the households and 789 from the gallery forests) were captured from the two study areas. The specimens were distributed across 11 genera and 30 species (genus, subgenus and subtribe identifications were not considered for the number of registered species). Species richness was greater in Taquaruçu (n = 24) than in downtown Palmas (n = 22). The most frequent species was *Ny. whitmani* (50.6%), followed by *Ev. carmelinoi* (7.53%), *Bichromomyia flaviscutellata* (7.27%), *Psathyromyia hermanlenti* (6.8%), *Lu. longipalpis* (6.2%), and *Ev. walkeri* (4.5%). The other species represented 15% of the total number of sand fly species captured (Table I). Fifteen species were common in both locations. Six species were found only in Palmas, and nine species were detected only in Taquaruçu (Table I). Sand flies were more frequent in the HUs in November (the rainy season) and in the gallery forests in July (the dry season), as shown in Tables II-III.

**TABLE I:** Total number of Phlebotominae specimens collected in the Taquaruçu district and downtown area of Palmas, Tocantins, Brazil, in July (the dry season) and November (the rainy season) of 2014

Species	Downtown Palmas		Taquaruçu		Total
	
	Dry month	Rainy month	Dry month	Rainy month	
*Bichromomyia flaviscutellata*	44	3	62	2	111
*Brumptomyia brumpti*	0	0	2	0	2
*Brumptomyia* sp.	1	0	6	2	9
*Evandromyia carmelinoi*	19	22	15	59	115
*Evandromyia evandroi*	1	0	2	11	14
*Evandromyia lenti*	5	2	1	24	32
*Evandromyia sallesi*	0	0	6	13	19
*Evandromyia saulensis*	7	1	0	0	8
*Evandromyia termitophila*	3	2	0	4	9
*Evandromyia walkeri*	15	35	8	11	69
*Lutzomyia longipalpis*	31	54	6	4	95
Lutzomyiina sp.	0	1	0	0	1
*Micropygomyia longipennis*	5	2	2	2	11
*Micropygomyia rorotaensis*	3	4	0	11	18
*Micropygomyia* sp.	0	0	1	0	1
*Micropygomyia villelai*	3	0	2	0	5
*Nyssomyia antunesi*	0	5	0	0	5
*Nyssomyia whitmani*	177	216	323	57	773
*Pintomyia christenseni*	0	0	16	4	20
*Pintomyia damascenoi*	0	0	1	0	1
*Pressatia choti*	0	0	7	1	8
*Pressatia* sp.	0	0	2	0	2
*Psathyromyia abonnenci*	1	0	0	0	1
*Psathyromyia aragaoi*	0	0	22	4	26
*Psathyromyia brasiliensis*	0	2	0	0	2
*Psathyromyia campbelli*	1	0	0	0	1
*Psathyromyia campograndensis*	0	0	3	2	5
*Psathyromyia hermanlenti*	24	3	78	0	105
*Psathyromyia lutziana*	0	2	6	0	8
*Psathyromyia pradobarrientosi*	1	0	4	0	5
*Psathyromyia punctigeniculata*	1	0	0	0	1
*Psathyromyia (Psathyromyia)* sp.	0	1	13	1	15
*Psychodopygus davisi*	0	0	1	3	4
*Psychodopygus hirsutus*	0	0	1	0	1
*Sciopemyia sordellii*	4	3	13	5	25

Total	346	358	603	220	1527

**TABLE III:** Phlebotominae specimens recorded in three environments in the city of Palmas, Tocantins, Brazil, in the rainy season (November) of 2014

Species	Downtown Palmas			Taquaruçu			Total
	
	Intra	Peri	Forest	Intra	Peri	Forest	
*Bichromomyia flaviscutellata*	0	1	2	0	1	1	5
*Brumptomyia* sp.	0	0	0	0	2	0	2
*Evandromyia carmelinoi*	2	18	2	1	56	2	81
*Evandromyia evandroi*	0	0	0	0	11	0	11
*Evandromyia lenti*	0	1	1	2	22	0	26
*Evandromyia sallesi*	0	0	0	2	11	0	13
*Evandromyia saulensis*	0	1	0	0	0	0	1
*Evandromyia termitophila*	0	2	0	0	4	0	6
*Evandromyia walkeri*	9	22	4	3	6	2	46
*Lutzomyia longipalpis*	12	44	0	0	2	0	58
Lutzomyiina sp.	0	0	1	0	0	0	1
*Micropygomyia longipennis*	0	0	2	2	0	0	4
*Micropygomyia rorotaensis*	2	2	0	6	4	1	15
*Nyssomyia antunesi*	0	4	1	0	0	0	5
*Nyssomyia whitmani*	4	212	0	0	56	1	273
*Pintomyia christenseni*	0	0	0	0	4	0	4
*Pressatia choti*	0	0	0	0	0	1	1
*Psathyromyia (Psathyromyia)* sp.	0	1	0	0	1	0	2
*Psathyromyia aragaoi*	0	0	0	0	3	1	4
*Psathyromyia brasiliensis*	0	2	0	0	0	0	2
*Psathyromyia campograndensis*	0	0	0	0	0	2	2
*Psathyromyia hermanlenti*	1	2	0	0	0	0	3
*Psathyromyia lutziana*	2	0	0	0	0	0	2
*Psychodopygus davisi*	0	0	0	0	0	3	3
*Sciopemyia sordellii*	1	2	0	2	3	0	8

Total	33	314	13	18	186	14	578

Intra: intradomestic environment; Peri: peridomestic environment.

In total, 285 of the 960 HP traps installed were positive, resulting in 29.7% capture success. Sand flies were captured in Shannon traps in both months in the four forest sites sampled (n = 6, 75% capture success). There was a significant difference in the proportion of positive HP traps between the months (July = 44.3%; November = 15%), between the habitats (HUs = 23.3%; forest = 36%), and between the locations (Taquaruçu = 33.5%; Palmas = 25.8%). Fisher’s exact test showed significant differences in all comparisons, with p < 0.01. The highest capture success rate was obtained in the forests in July (63.3%), a result which was much higher than that of the forests in November (8.7%). In the HUs, the proportions of positive traps in July and November were more similar: 25.4% and 21.2%, respectively.

In July, the relative abundance of sand flies was higher in gallery forests than in the HUs (85.2% vs. 24.8% in Taquaruçu and 71.7% vs. 28.3% in downtown Palmas). Species richness was higher in July in both Taquaruçu and downtown Palmas (20 species and 14 species, respectively) (Table II). The species with the highest rate of occurrence in the Taquaruçu gallery forests in this dry month were *Ny*. *whitmani* (47.9%), *Pa*. *hermanlenti* (11.8%), *Bi*. *flaviscutellata* (10.1%), *Pa*. *aragaoi* (3.5%), and *Pintomyia christenseni* (2.9%). As shown in Table II, the most frequent species in the gallery forests in downtown Palmas were *Ny*. *whitmani* (42.5%), *Bi*. *flaviscutellata* (12.7%), *Ev*. *walkeri* (4%), and *Pa*. *hermanlenti* (3.7%). Only three *Lu*. *longipalpis* specimens were found in the gallery forest in downtown Palmas.

In November, the number and richness of Phlebotominae captured in the gallery forests were low. Most of the species collected that month were found in the peridomestic sites in downtown Palmas, and species richness was similar between the peridomestic sites in Palmas and Taquaruçu (Table III). The most frequent species in the peridomestic environment in downtown Palmas in November was *Ny*. *whitmani* (59%), followed by *Lu*. *longipalpis* (11.9%), *Ev*. *walkeri* (6.1%), and *Ev*. *carmelinoi* (5%). Meanwhile, in the peridomestic environment in Taquaruçu, the most frequent species were *Ny*. *whitmani*, *Ev*. *carmelinoi* (25.6%), *Evandomyia lenti* (10%), *Evandromyia sallesi* (5%), and *Evandromyia evandroi* (4.6%) (Table III).

**Fig. 2:**
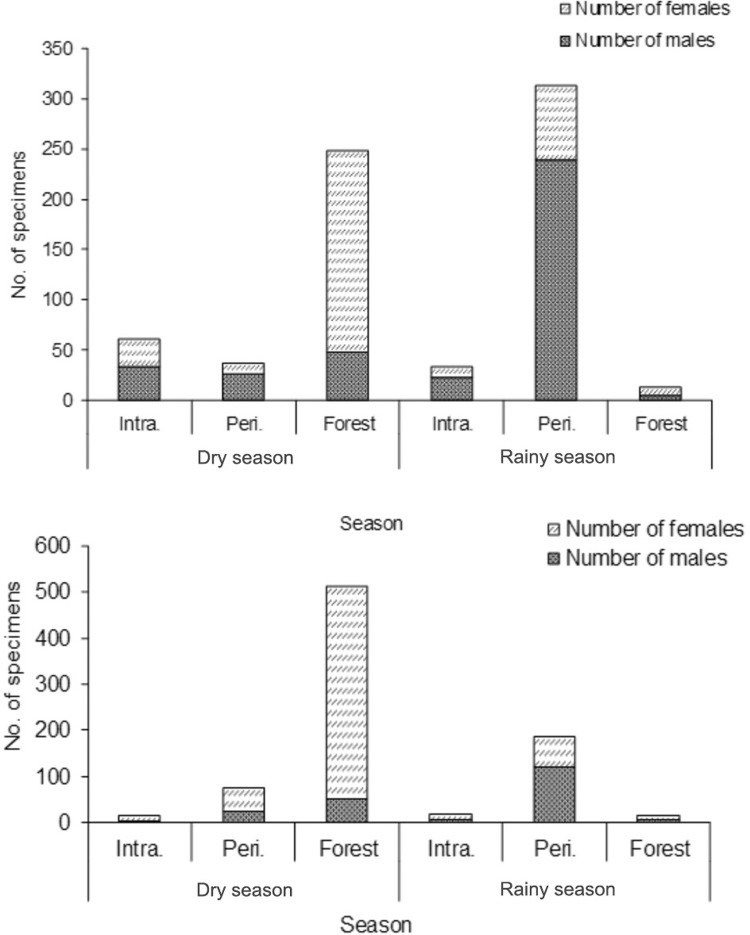
: abundance of male and female Phlebotominae specimens in three environments in Palmas (top) and Taquaruçu (bottom), Brazil, in July (the dry season) and November (the rainy season) of 2014. Intra: intradomestic environment; Peri: peridomestic environment; No: number.

The overall ratio of females to males (F:M) was 1.6:1. More females were captured than males in July (764 females versus 185 males, ratio = 4.1:1), though more males than females were captured in November (180 females versus 398 males, ratio = 0.4:1). More females were captured in the gallery forests in downtown Palmas in July (the dry month), while more males were captured in the peridomestic site in downtown Palmas in the rainy month (Fig. 2). In Taquaruçu in July, there were also more females in the gallery forests, unlike in November, in which more males were captured in the HUs of this district (Fig. 2).

**Fig. 3:**
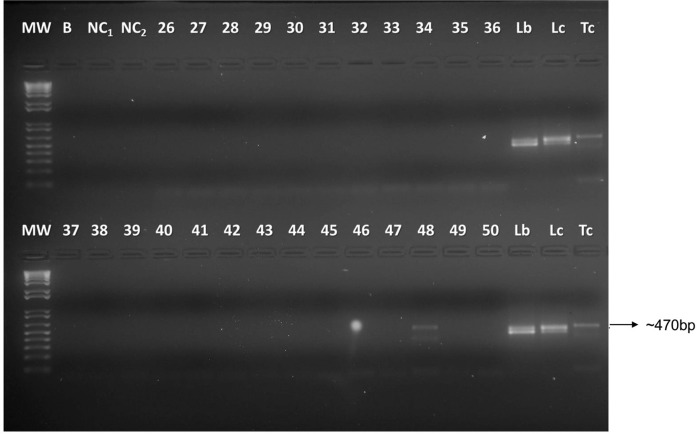
: identification of trypanosomatids in sand fly samples captured in Palmas, Tocantins, Brazil, in July 2014 based on polymerase chain reaction (PCR) directed to the SSU rDNA gene. 26 to 50: sandfly samples. MW: molecular weight marker; positive controls: Tc (*Trypanosoma cruzi*), Lc (*Leishmania infantum*), Lb (*Leishmania braziliensis*). NC: negative control; B: blank.

A total of 752 females grouped into 154 pools were analysed (Supplementary data). Of these, 15 pools exhibited positive amplification in the SSU (18S) rDNA gene (e.g. Fig. 3, GenBank accession numbers KY660706-KY660719). Only one of these samples were positive in the ITS1-PCR. *Leishmania* spp. DNA was detected in the sand fly species from both sample locations (Table IV); however, there were no significant differences in the proportions of pools positive for *Leishmania* spp. between these locations in either July (Fisher, p = 0.74) or November (Fisher, p = 1.0). In July, the proportion of phlebotomine pools positive for *Leishmania* spp. DNA were 17.6% in the HUs and 3.7% in the gallery forests. In November, the proportion of pools positive for *Leishmania* spp. DNA were 5.8% in the HUs and 28.5% in the forests. When the sampling in both months was considered, the proportion of Phlebotominae pools positive for *Leishmania* was 8/57 (14.0%) in the HUs and 6/84 (7.1%) in the forests (Fisher, p = 0.25).

**TABLE IV:** Trypanosomatids identified by sequencing of amplified SSU rDNA products detected in Phlebotominae specimens for the city of Palmas, Tocantins, Brazil, in July (the dry season) and November (the rainy season) of 2014

Nº	Species	Location	Habitat	BLASTN	Accession number	Identity
Dry month						
11	*Nyssomyia whitmani*	Taquaruçu	Forest	*Leishmania* sp.	gb|JX030135.1*	423/425 (99%)
13	*Ny. whitmani*	Taquaruçu	Forest	*Leishmania* sp.	gb|JX030135.1*	419/420 (99%)
16	*Ny. whitmani*	Taquaruçu	Forest	*Crithidia fasciculata*	gb|DQ182544.1	475/475 (100%)
48	*Ny. whitmani*	Taquaruçu	Intradomicile	*Leishmania* sp.	gb|JX030135.1*	397/398 (99%)
61	*Pintomyia christenseni*	Taquaruçu	Forest	*Trypanosoma* sp.	gb|JX853185.1*	470/470 (100%)
104	*Lutzomyia longipalpis*	Taquaruçu	Forest	*L. amazonensis*	JX030083.1	401/401 (100%)
107	*Lu. longipalpis*	Palmas	Intradomicile	*L. amazonensis*	JX030083.1	416/421 (99%)
114	*Ny. whitmani*	Palmas	Peridomicile	*L. infantum*	XR_001203206.1	425/429 (99%)
115	*Ny. whitmani*	Taquaruçu	Peridomicile	*L. amazonensis*	JX030083.1	427/428 (99%)
116	*Ny. whitmani*	Taquaruçu	Intradomicile	*L. amazonensis*	JX030083.1	429/430 (99%)
119	*Psathyromyia hermanlenti*	Taquaruçu	Intradomicile	*L. amazonensis*	JX030083.1	429/430 (99%)
Rainy month						
16	*Lu. longipalpis*	Palmas	Peridomicile	*L. amazonensis*	JX030083.1	426/429 (99%)
22	*Evandromyia walkeri*	Palmas	Forest	*L. amazonensis*	JX030083.1	429/430 (99%)
46	*Pi. christenseni*	Taquaruçu	Peridomicile	*L. amazonensis*	JX030083.1	429/430 (99%)
48	*Bichromomyia flaviscutellata*	Taquaruçu	Forest	*Leishmania* sp.	XR_001203206.1/ JX030135.1*	135/172 (78%)

BLASTN: basic local alignment search tool; Nº: number of pools; *: the sequence did not allow for species identification. The accession number reported indicates one of the sequences similar to the sequence obtained herein.

In Taquaruçu district in July, six *Ny*. *whitmani* pools were found to have DNA from the following trypanosomatid species: *Leishmania* spp. (n = 3; forests and HUs), *L. amazonensis* (n = 2; HU), and *Crithidia fasciculata* (n = 1; forest). In addition, one *Pi. christenseni* pool (n = 1; forest) was found to have *Trypanosoma* sp*.* DNA (Table IV). In this month, *Lu. longipalpis* and *Pa*. *hermanlenti* pools were found to have *L*. *amazonensis* DNA (Table IV). In downtown Palmas, *L. infantum* and *L*. *amazonensis* DNA was detected in *Ny. whitmani* pools and *Lu. longipalpis* pools, respectively (Table IV).

The minimum rates of infection of *Ny*. *whitmani* (number of positive pools/number of females analysed) by *L*. *amazonensis* and *L. infantum* in HUs in July (the dry month) were 7%, and 3%, respectively. In November (the rainy month), no *Leishmania*-infected *Ny*. *whitmani* was detected. The minimum rates of infection of *Lu. longipalpis* by *L*. *amazonensis* in HUs were 10% in November and 12.5% in July. In November, *Leishmania* sp. was detected in *Bi*. *flaviscutellata* and *L*. *amazonensis* was detected in *Ev. walkeri* and *Pi. christenseni* (Table IV).

## DISCUSSION

The presence of *Leishmania* spp. DNA in six Phlebotominae species from HUs and adjacent gallery forests in downtown Palmas and the Taquaruçu district suggests that the environments are potentially at risk for the *Leishmania* transmission. The results also reveal the role of gallery forests in maintaining *Leishmania* vectors in areas of the Brazilian savanna.

The number of species recorded in this study (30) was similar to the number reported by Machado et al. (2012), who registered 32 species. High Phlebotominae diversity has also been reported in studies performed in different cities in this state (Andrade-Filho et al. 2001, Vilela et al. 2011, 2013). Species such as *Ny. whitmani*, *Bi*. *flaviscutellata*, and *Lu. longipalpis* are likely to be involved in the transmission of *Leishmania* spp. in Palmas.

Species richness and Phlebotominae occurrence were higher in the rural area (Taquaruçu) than in downtown Palmas. Parallel findings were also reported by Cruz et al. (2013) in Bandeirantes, state of Paraná. Meanwhile, Vilela et al. (2011) also observed high species diversity in rural environments, but the abundance of sand flies was higher in urban environments. These results may be due to the presence of forests close to households and animal housing structures, as well as to the presence accumulated organic matter, all of which provides conditions that are conducive to the establishment of a Phlebotomine reproductive cycle (Quinnell & Dye 1994, Cruz et al. 2013).

Species richness was higher in July (the dry month) than in November (the rainy month) in both locations. Similar findings were reported by Pinheiro et al. (2013) in the state of Rio Grande do Norte and Moraes et al. (2015) in state of Maranhão. In the current study, the soil showed higher amount of organic matter in dry month (see Fig. in Supplementary data). This condition may have contributed to the higher population size in this month and, as a result, to the increase in permanent breeding sites. In the dry month, the preferred habitat among the sand fly specimens was the adjacent gallery forests, where higher numbers of females were captured. In November (the rainy month), most of the specimens captured were found in the peridomestic sites, and there were more males than females. The lower number of phlebotomines in gallery forests was observed in the rainy season when the soil was very soaked (see Fig. in Supplementary data). Probably this environmental condition disturbs the species development in the soil reducing number of breeding sites and consequently population size. The predominance of females over males in this study differed from the findings of other authors (Vilela et al. 2011, 2013, Cruz et al. 2013, Pinheiro et al. 2013), and this occurred probably due the food source or climatic changes. However, a detailed sex ratio study during a one or two-year period is necessary to test such hypothesis.

PCR has been used in many studies to detect *Leishmania* in Phlebotominae species (Quaresma et al. 2012, Vilela et al. 2013). It is more sensitive than dissection followed by parasitological examination, and it allows for the identification of parasites at the genus, subgenus, and/or species level (Pita-Pereira et al. 2011). The nested PCR technique using SSU rDNA is more sensitive in the detection of trypanosomatids than PCR with ITS1 as a target (El Tai et al. 2000), a factor which would explain the unique positive sample detected using the ITS1 marker. The rates of infection by *Leishmania* in vectors are generally low in nature (Paiva et al. 2006). The average rates remained below 3%, and have rarely reached 10% when evaluated by dissection or PCR (Missawa et al. 2010). Based on this, some of our rates were considered high.

In Taquaruçu, *Leishmania* spp. DNA detected in the *Ny. whitmani* may be from *L. braziliensis*, as *Ny. whitmani* is an important vector of the parasite (Rangel & Lainson 2009). *L. amazonensis* DNA was detected for the first time in *Ny. whitmani*, *Pa*. *hermanlenti*, and *Pi. christenseni* from this location. It is known that *Ny*. *whitmani* can be infected experimentally with *L. amazonensis* and may even be a vector of diffuse cutaneous leishmaniasis (Fonteles et al. 2016). Infection by *Leishmania* sp. detected in *Bi*. *flaviscutellata* from this site may be caused by *L. amazonensis*, given the known association between these species (Rangel & Lainson 2009). *Crithidia fasciculata* DNA was detected for the first time in *Ny*. *whitmani* from this district, as was DNA of *Trypanosoma* sp. in *Pi. christenseni*. In downtown Palmas, DNA of *L*. *infantum* was detected in *Ny*. *whitmani*, and the presence of *L. amazonensis* was detected in *Ev*. *walkeri* for the first time. Infections of *Ny. whitmani* by *L*. *infantum* were described in state of Minas Gerais, but the biological interactions involved in this infection need to be investigated to determine the role of this vector in parasite transmission (Margonari et al. 2010). The finding of *L*. *infantum* DNA in *Lu. longipalpis* at both locations was expected, but *L*. *amazonensis* DNA was found in this sand fly species. Experiments have revealed the ability of this vector to transmit *L*. *amazonensis* to hamsters (da Silva et al. 1990), and other studies have detected *L*. *amazonensis* in *Lu. longipalpis* (Paiva et al. 2006, Savani et al. 2009).

The first records of *L. amazonensis* DNA in *Ny*. *whitmani*, *Pi. christenseni*, *Pa*. *hermanlenti*, and *Ev. walkeri* in the city of Palmas suggest the possible involvement of these species in the transmission of the diffuse cutaneous form of the disease in this city, where 169 cases of ACL were reported in recent years (data obtained in cooperation with the Municipal Secretariat of Health). However, the finding of *Leishmania* DNA in sand fly species is not the only condition required to declare it a vector. Species distribution must also coincide with that of the disease in humans; the insect must also be found to be infected in peridomestic and domestic environments; it must feed regularly on humans and other hosts (Killick-Kendrick 1999), and the interaction between the vector and hosts must be analysed in mathematical models (Ready 2013). However, these reports will allow for a better understanding of the epidemiology of ACL in Palmas, and will also help government health agencies to define the best surveillance and control strategies for this zoonosis.

Though the small number of specimens captured, *Lu. longipalpis* was commonly found in the domestic environments in both downtown Palmas and Taquaruçu. Unlike in Taquaruçu, *Ny. whitmani* was more common in the HUs than in the gallery forests in downtown Palmas, a finding which evidences that this species is able to adapt to environmental changes and to domiciliary environments (Vilela et al. 2011). Furthermore, *Leishmania* spp. DNA was detected in both species in both study areas. It is therefore possible that *L. infantum* and *L. amazonensis* are transmitted by *Ny. whitmani* and that *L. amazonensis* is also transmitted by *Lu. longipalpis* in both locations.

The results indicate that the gallery forests play an important role in maintaining Phlebotomine populations in areas of the Brazilian savanna, particularly during the dry month, when their frequency decreases in household units. *Ny*. *whitmani*, the main vector of ACL, was frequent in the gallery forests and household units of the study areas, as was *Lu. longipalpis*, a vector of VL. However, *Lu. longipalpis* was rare in the gallery forests, suggesting that the adjacent forests are more important habitats for ACL vectors than VL vectors, and that *L. infantum* cycles are largely independent of these forest environments. The new records of Phlebotominae infections by *Leishmania* species and other trypanosomatid species presented herein provide increased knowledge on parasite-vector relations in areas of the Brazilian savanna.

**TABLE II:** Phlebotominae specimens recorded in three environments in the city of Palmas, Tocantins, Brazil, in the dry season (July) of 2014

Species	Downtown Palmas			Taquaruçu			Total
	
	Intra	Peri	Forest	Intra	Peri	Forest	
*Bichromomyia flaviscutellata*	0	0	44	0	1	61	106
*Brumptomyia brumpti*	0	0	0	1	0	1	2
*Brumptomyia* sp.	0	0	1	0	0	6	7
*Evandromyia carmelinoi*	8	7	4	0	15	0	34
*Evandromyia evandroi*	0	0	1	0	0	2	3
*Evandromyia lenti*	1	1	3	0	1	0	6
*Evandromyia sallesi*	0	0	0	0	3	3	6
*Evandromyia saulensis*	0	0	7	0	0	0	7
*Evandromyia termitophila*	2	1	0	0	0	0	3
*Evandromyia walkeri*	0	1	14	0	0	8	23
*Lutzomyia longipalpis*	19	10	2	0	5	1	37
*Micropygomyia longipennis*	0	0	5	1	1	0	7
*Micropygomyia rorotaensis*	0	0	3	0	0	0	3
*Micropygomyia* sp.	0	0	0	0	0	1	1
*Micropygomyia villelai*	1	1	1	0	1	1	5
*Nyssomyia whitmani*	17	13	147	4	30	289	500
*Pintomyia christenseni*	0	0	0	1	0	15	16
*Pintomyia damascenoi*	0	0	0	0	0	1	1
*Pressatia choti*	0	0	0	0	4	3	7
*Pressatia* sp.	0	0	0	0	0	2	2
*Psathyromyia abonnenci*	0	1	0	0	0	0	1
*Psathyromyia aragaoi*	0	0	0	0	1	21	22
*Psathyromyia campbelli*	1	0	0	0	0	0	1
*Psathyromyia campograndensis*	0	0	0	0	0	3	3
*Psathyromyia hermanlenti*	9	2	13	1	6	71	102
*Psathyromyia lutziana*	0	0	0	1	0	5	6
*Psathyromyia pradobarrientosi*	0	0	1	0	0	4	5
*Psathyromyia punctigeniculata*	1	0	0	0	0	0	1
*Psathyromyia (Psathyromyia)* sp.	0	0	0	2	2	9	13
*Psychodopygus davisi*	0	0	0	0	0	1	1
*Psychodopygus hirsutus*	0	0	0	0	0	1	1
*Sciopemyia sordellii*	2	0	2	4	4	5	17

Total	61	37	248	15	74	514	949

Intra: intradomestic environment; Peri: peridomestic environment.
