# Supplementary material for: The role of gallery forests in maintaining Phlebotominae populations: potential Leishmania spp. vectors in the Brazilian savanna
Source: Mem Inst Oswaldo Cruz. 2017 Oct;112(10):681–91. doi: 10.1590/0074-02760170126 (PMC5607517; doi:10.1590/0074-02760170126)
Supplement: Supplementary file 1 [file 0074-0276-mioc-112-10-0681-suppl01.pdf]

TABLE I

Phlebotominae specimens collected from household units (HUs) and gallery forests in Palmas, Tocantins, Brazil, in July (the dry season) of 2014 and determined to be positive for infection based on SSU rDNA and ITS1 markers

	Household Units				Gallery forests			
Species	Nº*	Pools	Nested SSU-PCR	ITS1-PCR	Nº*	Pools	Nested SSU-PCR	ITS1-PCR
Bichromomyia flaviscutellata	1	1	0	0	74	12	0	0
Brumptomyia sp.	0	0	0	0	3	1	0	0
Evandromyia carmelinoi	15	5	0	0	3	2	0	0
Evandromyia saulensis	0	0	0	0	5	1	0	0
Evandromyia walkeri	0	0	0	0	15	4	0	0
Lutzomyia longipalpis	8	6	1	0	2	2	1	0
Micropygomyia villelai	3	3	0	0	0	0	0	0
Nyssomyia whitmani	30	14	4	1	380	42	3	0
Pintomyia christenseni	0	0	0	0	11	2	1	0
Psathyromyia aragaoi	0	0	0	0	4	1	0	0
Psathyromyia hermanlenti	4	3	1	0	59	9	0	0
Psychodopygus davisi	0	0	0	0	2	1	0	0
Sciopemyia sordellii	4	2	0	0	4	2	0	0
Total	65	34	6	1	562	79	5	0

*: number of specimens.

TABLE II

Number of Phlebotominae samples collected from household units (HUs) and gallery forests in Palmas, Tocantins, Brazil, in November (the rainy season) of 2014 and determined to be positive for infection based on SSU rDNA and ITS1 markers

	Household Units				Gallery forests			
Species	Nº*	Pools	Nested SSU-PCR	ITS1-PCR	Nº*	Pools	Nested SSU-PCR	ITS1-PCR
Bichromomyia flaviscutellata	0	0	0	0	2	2	1	0
Evandromyia carmelinoi	25	6	0	0	2	1	0	0
Evandromyia evandroi	5	1	0	0	0	0	0	0
Evandromyia lenti	5	2	0	0	0	0	0	0
Evandromyia sallesi	3	1	0	0	0	0	0	0
Evandromyia termitophila	2	1	0	0	0	0	0	0
Evandromyia walkeri	10	6	0	0	2	1	1	0
Lutzomyia longipalpis	10	3	1	0	0	0	0	0
Micropygomyia rorotaensis	4	2	0	0	0	0	0	0
Nyssomyia whitmani	48	9	0	0	1	1	0	0
Pintomyia christenseni	3	3	1	0	0	0	0	0
Psathyromyia campograndensis	0	0	0	0	2	1	0	0
Psychodopygus davisi	0	0	0	0	1	1	0	0
Total	115	34	2	0	10	7	2	0

*: number of specimens.

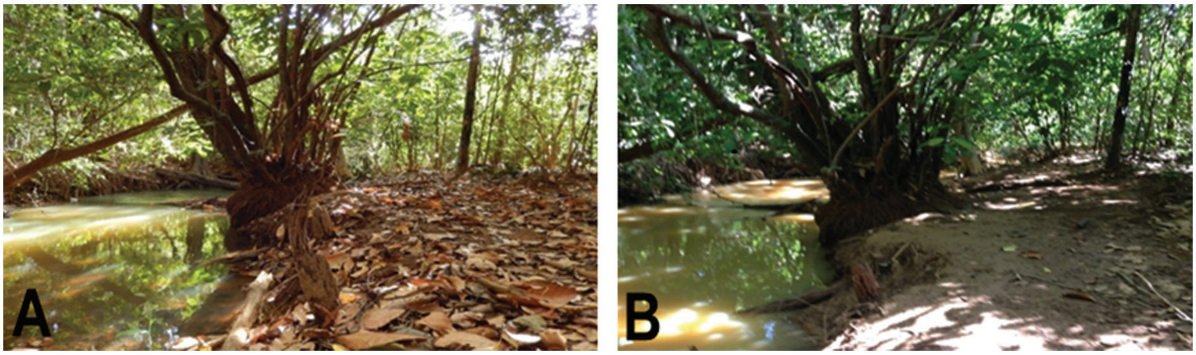

Photographs showing difference in the gallery forests during dry and rainy months in Palmas, Tocantins, Brazil. (A) July (dry month) with presence of dry leaves and higher organic matter in the soil. (B) November (rainy month).
